# Study Design in Clinical and Epidemiologic Research: Observational, Experimental, and Beyond

**DOI:** 10.1111/jre.70105

**Published:** 2026-03-11

**Authors:** Gustavo G. Nascimento, Ignacio Leiva‐Escobar, Maria Clotilde Carra, Sebastian‐Edgar Baumeister

**Affiliations:** ^1^ School of Dentistry University of Utah Salt Lake City Utah USA; ^2^ Institute of Health Services Research in Dentistry University of Münster Münster Germany; ^3^ Department of Translational Medicine University of Ferrara Ferrara Italy; ^4^ METHODS Team, CRESS, Université Paris Cité Paris France

**Keywords:** epidemiology, longitudinal studies, research design

## Abstract

Population‐based epidemiology and clinical research are complementary and mutually reinforcing: Epidemiology quantifies disease incidence/prevalence and elucidates environmental and social determinants in populations, whereas clinical research evaluates biomarkers, therapeutics, and prognostic outcomes in patients; their convergence underpins translational science, comparative effectiveness, real‐world evidence, and precision dentistry.

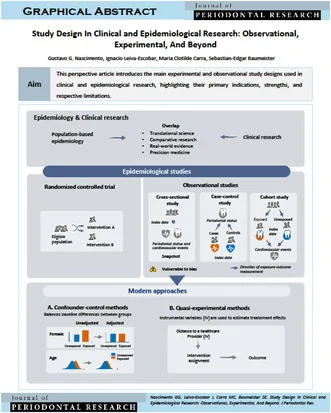

## 1 Introduction

Clinical research encompasses a broad range of scientific approaches aimed at understanding disease etiology, prevention, diagnosis, prognosis, and treatment. Epidemiology plays a central role within this landscape by providing population‐based methods to quantify and monitor disease occurrence, identify risk factors and causes, as well as evaluate interventions. Importantly, epidemiological research is not limited to individuals with established disease but often includes healthy individuals at risk of developing disease, thereby extending beyond the boundaries of clinical epidemiology narrowly defined [1].

This perspective article introduces the main experimental and observational study designs used in clinical and epidemiological research, highlighting their primary indications, strengths, and respective limitations. Rather than providing an exhaustive methodological compendium, the aim is to introduce readers to the conceptual logic underlying different designs and to prime them for the more specialized articles in this methodological series (Figure 1).

**FIGURE 1:**
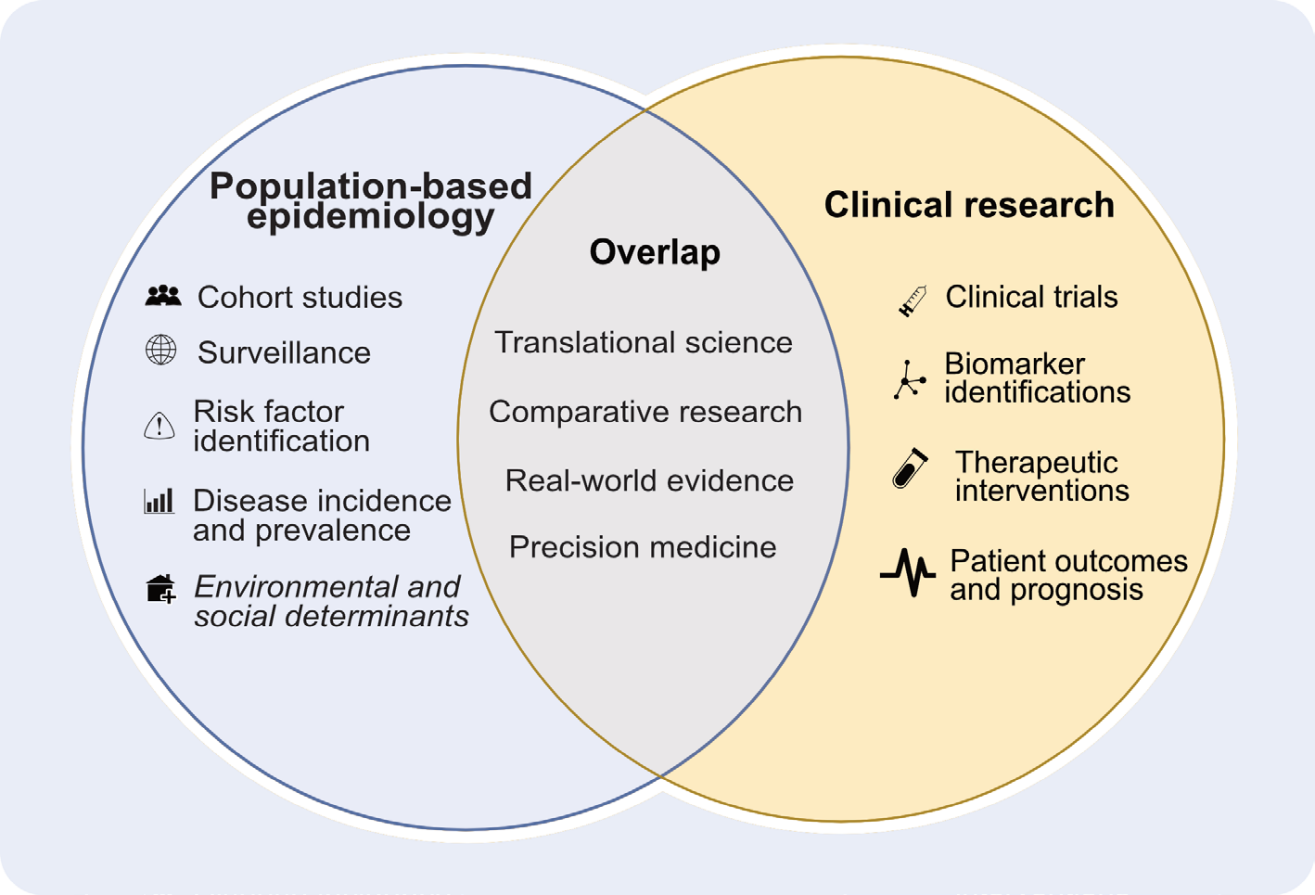
Conceptual overlap between population‐based epidemiology and clinical research. Population‐based epidemiology focuses on cohort studies, surveillance, risk factor identification, disease incidence and prevalence, and environmental and social determinants, whereas clinical research emphasizes clinical trials, biomarker identification, therapeutic interventions, and patient outcomes and prognosis. Their intersection encompasses translational science, comparative research, real‐world evidence, and precision medicine, illustrating how these disciplines complement and inform one another.

To illustrate how design choice influences interpretation, feasibility, and susceptibility to bias, we use a single running example throughout this article: the association between periodontitis and cardiovascular disease.

## 2 A Brief Introduction to Epidemiology

Epidemiology is the study of the distribution and determinants of health‐related states or events in defined populations, and the application of this knowledge to prevent and control health problems. Its broad scope and versatile methods span many domains of public health and clinical medicine.

Epidemiological research can be grouped into three general aims: descriptive, causal, and predictive [2, 3, 4]. Descriptive epidemiology quantifies the occurrence and distribution of health states or exposures across person, place, and time; its core functions include surveillance, burden estimation, characterization of natural history, and trend monitoring. Causal research estimates the effects of exposures or interventions on outcomes, either through experiments or by addressing “what if” questions that inform disease mechanisms and evidence‐based prevention and treatment [3, 5]. Predictive research focuses on diagnostic and prognostic modeling [6]. Diagnostic models typically use cross‐sectional data to estimate the current probability of disease, whereas predictive and prognostic models use longitudinal data to estimate the risk of future outcomes [6, 7]. These studies primarily evaluate model performance and clinical utility to support decision‐making.

Epidemiological studies can be designed as experimental or nonexperimental (i.e., observational) studies (Table 1). While causal inference has traditionally been driven by experimental studies, such as clinical trials, observational studies also play a crucial role, depending on the research question, data quality, and feasibility. In the following sections, we offer a concise overview of the most common epidemiological study designs (Figure 2).

**TABLE 1:** Epidemiological study designs.

Epidemiological research type	Objectives	Study designs	Strengths	Limitations
Descriptive research	Characterizes the distribution of diseases, exposures, or health‐related states in populations, without attempting to explain causal mechanisms. Primarily hypothesis‐generating	Cross‐sectional studies Cohort studies Surveillance studies Ecological studies	Simple to conduct Provides essential epidemiological metrics Identifies patterns and trends	Cannot infer causality Vulnerable to confounding Limited explanatory power
Causal research	Evaluates whether an exposure or intervention causes an outcome, aiming to estimate causal effects under explicit assumptions	Randomized controlled trials Quasi‐experiments Cohort studies Case–control studies	Supports causal inference Informs prevention and intervention strategies	Requires strong assumptions Resource‐intensive Susceptible to bias especially in observational designs
Predictive research	Develops or validates models to predict diagnosis or future outcomes or risks (prognosis) in individuals or populations, regardless of causality. Focuses on accuracy and calibration rather than on explanation	Cross‐sectional studies (diagnostic models and validation studies) Prognostic cohort studies Machine learning studies	Supports risk stratification and decision‐making Accommodates complex data	Limited causal interpretability Limited generalizability

**FIGURE 2:**
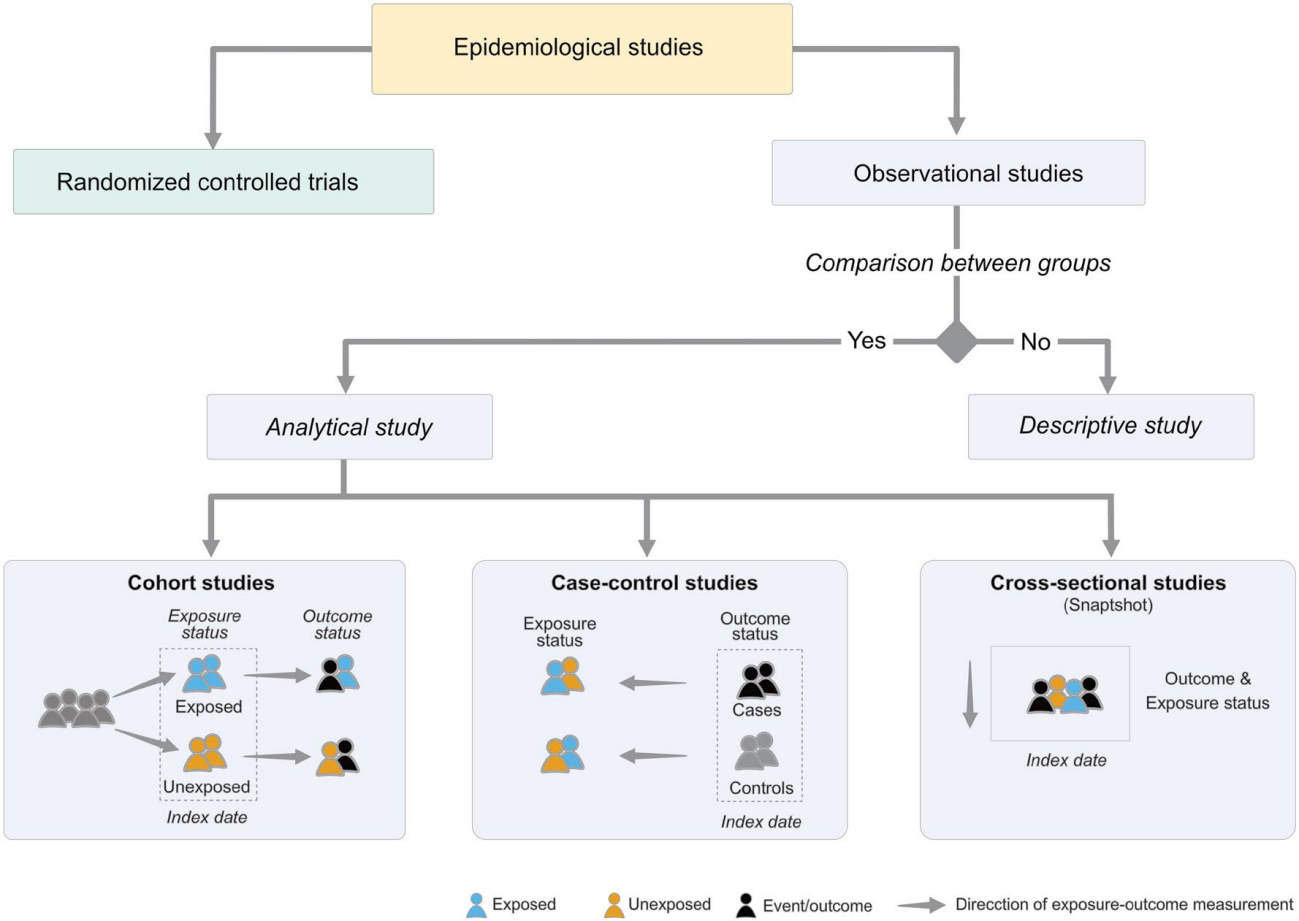
Overview of epidemiological studies and subclassifications, including randomized controlled trials and observational studies.

## 3 Experimental Study Designs

### 3.1 Randomized Controlled Trials

Randomized controlled trials (RCTs) are designed to infer causality by minimizing selection and confounding biases through randomization, strict eligibility criteria, and blinding. Randomization is expected to remove confounding by de‐coupling the probability of receiving a particular treatment from patients' characteristics. Without randomization, age, disease severity, or comorbidity may affect the choice of therapy. These characteristics and not the experimental treatment may then account for any apparent differences in risk and outcomes in a comparison of treatment groups. Such a situation would represent confounding. While RCTs are often said to “eliminate” confounding, it is more accurate to say that they *minimize* it: If core principles are not rigorously followed, methodological flaws can still jeopardize the validity of their findings.

RCTs can adopt several design configurations, selected according to the research question, the nature of the intervention, and biological and practical constraints. Parallel‐arm RCTs represent the most widely used design, in which participants are randomly allocated to two or more intervention groups and followed concurrently; this approach minimizes contamination between interventions and is particularly appropriate when treatment effects are irreversible or long‐lasting. Crossover RCTs involve the sequential administration of two or more interventions to the same participant in randomized order, separated by adequate washout periods; by allowing each individual to serve as their own control, this design enhances statistical efficiency but requires the assumption of no carryover effects, which may limit its applicability to reversible outcomes. Finally, typical of dental research, split‐mouth RCTs randomize different interventions to separate sites within the same oral cavity (e.g., contralateral quadrants or teeth), thereby controlling for interindividual variability and increasing power with smaller sample sizes; however, this design is susceptible to intraoral carryover effects and requires careful consideration of anatomical independence and outcome assessment. The choice among these designs should be guided by biological plausibility, risk of interference between interventions, and the validity of underlying methodological assumptions to ensure robust and interpretable causal inference.

RCTs are not without limitations. Large, well‐conducted trials are costly—often running into millions of dollars. External validity is another major concern, as it is not always clear to what extent the findings can be extrapolated to broader patient populations. Strict eligibility criteria enhance internal validity and improve feasibility, adherence to the study protocol, while reducing loss to follow‐up, but they also limit generalizability. Moreover, treatment effects in RCTs may be overestimated due to small sample size (e.g., vibration of effects [8]) as well as phenomena like the Hawthorne effect, where participants modify their behavior because they are under observation.

Finally, some exposures cannot be randomized for ethical or biological reasons. Individuals cannot be intentionally exposed to noxious substances (e.g., smoking), induced to adopt harmful behaviors or conditions (e.g., become obese), or assigned to a particular socioeconomic position, or sex. In such circumstances, clinicians must rely on other types of study designs to inform practice.

## 4 Non‐Experimental (Observational) Study Designs

### 4.1 Cross‐Sectional Studies

We begin with cross‐sectional studies, which are often the first design encountered in population‐based and clinical research. Cross‐sectional studies collect information on exposures and outcomes at a single point in time and are often described as a “snapshot” of a population. Their primary strength lies in estimating disease prevalence and population burden, making them particularly useful for surveillance, needs assessment, and hypothesis generation. However, because exposure and outcome are measured simultaneously, temporal ordering cannot be established, and reverse causation is a major concern. As a result, cross‐sectional studies are generally unsuitable for causal inference.

For example, a cross‐sectional analysis using data from the National Health and Nutrition Examination Survey (NHANES) may compare the prevalence of self‐reported cardiovascular disease among individuals with and without periodontitis at the time of examination, given that both cardiovascular disease and periodontitis are measured simultaneously [9]. Such an analysis can quantify the population burden of comorbidity and identify disparities, but it cannot determine whether periodontitis preceded cardiovascular disease or whether the observed association reflects shared risk factors such as smoking or socioeconomic position. For further details about cross‐sectional studies, including their applications and limitations, please refer to Baumeister et al. [10] published in this Special Issue.

### 4.2 Cohort Studies

When temporal ordering between exposure and outcome is essential, cohort studies provide a natural next step. The starting point of a cohort study involves identifying groups within a population based on their exposure levels, typically separating them into exposed and unexposed cohorts. Unlike randomized trials, where exposure is assigned experimentally, observational cohort studies define groups based on naturally occurring exposures. A fundamental requirement is that all participants must be free of the outcome of interest and at risk of developing it at the start of the study. Additionally, regardless of whether the study is prospective (coinciding with the observation period) or retrospective (utilizing existing records), the measurement of exposure must always precede the occurrence of the outcome. During the observation period, cohort members are traced until the outcome occurs, or until death, emigration, dropout, or the study concludes.

Although cohort studies are well suited for examining disease incidence, prognosis, and multiple outcomes associated with a given exposure, they are inefficient for rare exposures, as very large sample sizes and extended follow‐up are required to accrue sufficient exposed individuals and outcome events. Hence, cohort studies can be time‐consuming, costly, and vulnerable to loss to follow‐up (attrition), which may introduce bias if participants who drop out differ systematically from those who remain. Confounding also remains a concern, as exposure is not randomly assigned; therefore, careful design and analytic adjustment are essential.

Using the running example, a cohort study would enroll adults without prior cardiovascular disease, classify them according to periodontal status at baseline, and follow them for incident cardiovascular events. This design establishes temporal ordering and allows estimation of incidence rates and relative risks, but valid inference depends on careful measurement and control of pre‐exposure confounders such as smoking, diabetes, and access to health care. Further information on cohort studies, including their applications and limitations, can be found in a dedicated article focused on this study design published in this Special Issue.

### 4.3 Case–Control Studies

When outcomes are rare or have long induction periods, cohort studies may be impractical, and case–control studies offer an efficient alternative. The cohort design may prove impractical or unfeasible under certain conditions, specifically if the outcome of interest is rare; the relationship between the exposure and the outcome involves a long induction period; or the source population is large, open, and lacks a suitable database. In such instances, the case–control study serves as an efficient alternative. This design should be conceptually understood by imagining a hypothetical cohort study within a population. However, instead of assembling exposed and unexposed cohorts and waiting for events to occur, investigators ascertain events of interest that have already accumulated in the study population over time (cases) and take a sample of the underlying population to serve as controls. Information regarding exposure is then obtained for both the sampled cases and controls, specifically referencing the period before the outcome occurrence.

Cases are defined as events of interest arising during the observation period and can be identified either prospectively as they arise or retrospectively after the period ends. The date of the case occurrence is referred to as the index date. The selection of controls is critical; they must be selected from persons who would have been counted as a case had they experienced the event of interest. Furthermore, controls should be representative of the exposure distribution in the study population and must be sampled independently of their exposure status.

Case–control studies can assess many potential risk factors for a given disease and often serve as a first step in identifying associations worthy of further investigation. Their main limitations relate to selection and information biases: Choosing appropriate controls can be challenging, and retrospective assessment of exposures may be affected by incomplete records or differential recall between cases and controls. As a result, case–control studies typically estimate odds ratios rather than direct risks, and their findings must be interpreted with attention to potential bias and confounding. Confusing case–control studies with case–non‐case comparisons derived from cross‐sectional data can lead to incorrect interpretations, because the comparison group was chosen based on the absence of the outcome.

Applied to the running example, a case–control study would identify individuals with newly diagnosed (incident) cardiovascular disease as cases and sample controls from the population at risk at the same time. Prior periodontal status would then be assessed for both groups. This approach is efficient for rare cardiovascular outcomes but is vulnerable to selection bias and exposure misclassification, particularly when periodontal status is assessed retrospectively.

### 4.4 Case Series

Case series describe outcomes among a group of individuals sharing a clinical condition or intervention, without a comparison group. Although they may be useful for hypothesis generation or describing novel clinical phenomena, they do not permit estimation of disease frequency or causal effects. For this reason, case series are not considered epidemiological study designs in this article and are conceptually distinct from cohort studies, which explicitly compare outcome occurrence across exposure groups over time.

## 5 Contemporary Approaches to Study Design and Data Analysis

Traditional observational designs have yielded landmark findings, such as Doll and Hill's case–control study linking smoking to lung cancer and the Framingham Heart Study, which identified cardiovascular risk factors [11, 12]. For this reason, cross‐sectional, cohort, and case–control studies remain an excellent starting point for clinical researchers in the medical and dental fields. Nevertheless, observational studies are vulnerable to systematic errors (biases) that can lead to misleading conclusions. Classic examples include the apparent strong protective effects of hormone replacement therapy against coronary heart disease and of statins against cancer—effects that vanished in subsequent large RCTs [13]. These discrepancies often arose from inadequate control of confounding and, importantly, from misdefining the start of follow‐up (time zero), introducing so‐called immortal time bias by creating periods during which the outcome cannot occur by design, thereby making treatments appear artificially protective.

Over the past two decades, methodological advances have therefore focused on reducing confounding and selection biases in observational data. Modern approaches to minimizing bias can be broadly divided into confounder‐control methods and quasi‐experimental methods [14] (Figure 3). Regardless of the method applied to minimize biases, the use of directed acyclic graphs (DAGs) is of utmost importance in modern epidemiology. Briefly, DAGs provide an intuitive yet rigorous framework for communicating causal questions in clinical and epidemiologic research. Further details on DAGs can be found elsewhere [15]. Confounder‐control methods seek to balance baseline differences between exposed and unexposed groups using design and analytic strategies (such as regression adjustment or inverse probability weighting) (Figure 3A). In contrast, quasi‐experimental designs exploit natural or policy‐driven “as‐if random” sources of treatment variation (e.g., regional prescribing preferences or calendar‐time cutoffs) that affect treatment receipt but are otherwise unrelated to the outcome (Figure 3B). In other words, quasi‐experimental designs exploit sources of variation that approximate randomization.

**FIGURE 3:**
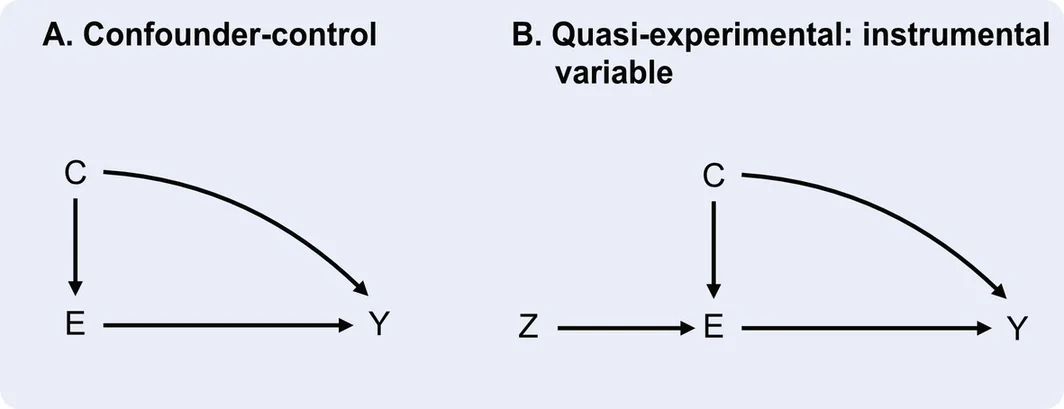
Approaches to study design and data analysis using observational data. (A) Directed acyclic graph (DAG) depicting the confounder‐control approach, where E represents the exposure, Y the outcome, and C a given set of confounders, which should be adjusted for in the analysis. (B) DAG depicting the use of a quasi‐experimental design, in this case, the application of instrumental variables, where Z denotes the instrument, E the exposure, Y the outcome, and C a given set of confounders.

A key evolution in confounder‐control cohort studies is the emphasis on starting follow‐up at treatment initiation (time zero), measuring covariates before exposure, and clearly defining treatment strategies [16, 17]. This practice avoids immortal time bias, allows detection of early treatment effects, and better handles treatment non‐persistence. Among these advances, the target trial emulation framework has gained particular traction [13]. It encourages researchers to explicitly design their observational study as an emulation of the RCT that would ideally answer the same question. Investigators prespecify eligibility criteria, treatment strategies, assignment procedures, time zero, outcome definitions, causal contrast (intention‐to‐treat or per‐protocol), and analysis plan, then map these components onto available observational data. This structured approach minimizes common biases, improves transparency, and clarifies assumptions. When combined with appropriate statistical methods (e.g., inverse probability weighting) and sensitivity analyses, target trial emulation offers dental researchers a rigorous, reproducible way to estimate treatment effects from observational data when randomized trials are infeasible—provided data quality is sufficient, and assumptions are clearly stated and tested.

Using the running example, a hypothetical trial would specify eligibility criteria (e.g., adults without prior cardiovascular disease), treatment strategies (e.g., periodontal treatment strategies), treatment assignment, outcome definitions, and start and end of follow‐up. Explicitly defining these components clarifies the causal question and helps to correctly identify time zero (baseline), the time at which eligibility is met, and individuals are assigned to a treatment strategy, to avoid common biases, including immortal time bias. A dedicated article in this series provides an in‐depth treatment of time‐related biases and target trial emulation [18].

When a random or quasi‐random source of variation in treatment or exposure is available, this can be exploited as an instrumental variable (IV) to aid in the causal identification of the treatment effect [14]. The concept of IV originated in the field of economics in the 1920s [19], while foundational work on the method's mathematical properties in the 1990s led to the awarding of the Nobel Prize in 2021 [20]. A classical application of IV analysis involves using randomized group assignment in a clinical trial to estimate the causal effect of the treatment received (adjusting for noncompliance) [21]. In the medical literature, a prominent example utilizes the differential distance to a healthcare provider as an IV for treatment intensity to estimate its effect on cardiovascular outcomes [22].

## 6 Final Remarks

This article is intended to orient readers to the conceptual logic and appropriate use of common study designs rather than to provide detailed guidance on statistical analysis or bias correction, which are addressed in other articles in this series. Instead, the optimal approach depends on the clinical question, the underlying causal structure, feasibility and ethics, and the strengths and limitations of the available data. Across designs, transparency in defining the estimand, specifying time zero, and articulating assumptions, including those about confounding, selection, and measurement, is essential for producing credible and clinically interpretable evidence.

By integrating classical epidemiologic principles with contemporary tools such as DAGs, target trial emulation, and quasi‐experimental approaches when appropriate, clinical researchers can improve the validity, reproducibility, and clinical relevance of their findings. Ultimately, stronger methods will help translate observational and experimental evidence into more reliable inferences for prevention, treatment, and patient care.

## Data Availability

Data sharing not applicable to this article as no datasets were generated or analysed during the current study.

## References

[1] D. A. Grimes and K. F. Schulz , “An Overview of Clinical Research: The Lay of the Land,” Lancet 359 (2002): 57–61. 10.1016/S0140-6736(02)07283-5 11809203

[2] K. Rothman , K. Huybrechts , and E. J. Murray , Epidemiology: An Introduction (Oxford University Press, 2024).

[3] T. L. Lash , T. J. VanderWeele , S. Haneuse , and K. J. Rothman , Modern Epidemiology (Wolters Kluwer, 2021). 10.1007/s10654-021-00778-w PMC8416883 34216355

[4] M. A. Hernán , J. Hsu , and B. Healy , “A Second Chance to Get Causal Inference Right: A Classification of Data Science Tasks,” Chance 32 (2019): 42–49.

[5] M. A. Hernan and J. M. Robin , Causal Inference: What if (Chapman & Hall/CRC, 2020).

[6] E. W. Steyerberg , Clinical Prediction Models (Springer, 2019).

[7] O. Efthimiou , M. Seo , K. Chalkou , T. Debray , M. Egger , and G. Salanti , “Developing Clinical Prediction Models: A Step‐By‐Step Guide,” BMJ 386 (2024): e078276. 10.1136/bmj-2023-078276 PMC11369751 39227063

[8] C. J. Patel , B. Burford , and J. P. Ioannidis , “Assessment of Vibration of Effects due to Model Specification Can Demonstrate the Instability of Observational Associations,” Journal of Clinical Epidemiology 68 (2015): 1046–1058. 10.1016/j.jclinepi.2015.05.029 PMC4555355 26279400

[9] P. I. Eke , L. Wei , G. O. Thornton‐Evans , et al., “Risk Indicators for Periodontitis in US Adults: NHANES 2009 to 2012,” Journal of Periodontology 87 (2016): 1174–1185. 10.1902/jop.2016.160013 PMC11370315 27367420

[10] S. E. Baumeister , T. Kocher , P. N. Papapanou , B. Holtfreter , and R. T. Demmer , “Cross‐Sectional Studies: Strengths, Limitations, and Methodological Considerations,” Journal of Periodontal Research (2026). 10.1111/jre.70063 PMC13665586 41487096

[11] S. E. Baumeister , I. Leiva‐Escobar , R. T. Demmer , et al., “Target Trial Emulation in Observational Research—Strengths, Limitations, and Methodological Considerations,” Journal of Periodontal Research (2026). 10.1111/jre.70095 PMC13665649 41793097

[12] D. Levy and W. B. Kannel , “Cardiovascular Risks: New Insights From Framingham,” American Heart Journal 116 (1988): 266–272. 10.1016/0002-8703(88)90099-3 3394638

[13] R. Doll and A. B. Hill , “Smoking and Carcinoma of the Lung; Preliminary Report,” British Medical Journal 2 (1950): 739–748. 10.1136/bmj.2.4682.739 PMC2038856 14772469

[14] M. A. Hernan , I. J. Dahabreh , B. A. Dickerman , and S. A. Swanson , “The Target Trial Framework for Causal Inference From Observational Data: Why and When Is It Helpful?,” Annals of Internal Medicine 178 (2025): 402–407. 10.7326/ANNALS-24-01871 PMC11936718 39961105

[15] E. C. Matthay , E. Hagan , L. M. Gottlieb , et al., “Alternative Causal Inference Methods in Population Health Research: Evaluating Tradeoffs and Triangulating Evidence,” SSM Popul Health 10 (2020): 100526. 10.1016/j.ssmph.2019.100526 PMC6926350 31890846

[16] J. C. Digitale , J. N. Martin , and M. M. Glymour , “Tutorial on Directed Acyclic Graphs,” Journal of Clinical Epidemiology 142 (2022): 264–267. 10.1016/j.jclinepi.2021.08.001 PMC8821727 34371103

[17] L. Cribb , M. Moreno‐Betancur , Z. Wu , R. Wolfe , M. Pase , and J. Ryan , “Moving Beyond the Prevalent Exposure Design for Causal Inference in Dementia Research,” Lancet Healthy Longevity 6 (2025): 100675. 10.1016/j.lanhl.2024.100675 39894022

[18] Q. L. Her , J. Rouette , J. C. Young , M. Webster‐Clark , and J. Tazare , “Core Concepts in Pharmacoepidemiology: New‐User Designs,” Pharmacoepidemiology and Drug Safety 33 (2024): e70048. 10.1002/pds.70048 PMC11588434 39586646

[19] J. H. Stock and F. Trebbi , “Retrospectives: Who Invented Instrumental Variable Regression?,” Journal of Economic Perspectives 17 (2003): 177–194.

[20] G. W. Imbens and J. D. Angrist , “Identification and Estimation of Local Average Treatment Effects,” Econometrica 62 (1994): 467–475.

[21] J. D. Angrist , G. W. Imbens , and D. B. Rubin , “Identification of Causal Effects Using Instrumental Variables,” Journal of the American Statistical Association 91 (1996): 444–455.

[22] M. McClellan , B. J. McNeil , and J. P. Newhouse , “Does More Intensive Treatment of Acute Myocardial Infarction in the Elderly Reduce Mortality? Analysis Using Instrumental Variables,” JAMA 272 (1994): 859–866. 8078163

